# Simultaneous UPLC Assay for Oxitropium Bromide and Formoterol Fumarate Dihydrate in Pressurized Metered Dose Inhaler Products for Chronic Obstructive Pulmonary Disease

**DOI:** 10.1093/jaoacint/qsad134

**Published:** 2023-12-18

**Authors:** Serdar Seckin, Serap Saglik Aslan

**Affiliations:** Istanbul University Institute of Health Sciences, Analytical Chemistry, 34126 Beyazıt, Istanbul, Türkiye; Istanbul University, Faculty of Pharmacy, Department of Analytical Chemistry, 34116 Beyazıt Istanbul, Türkiye

## Abstract

**Backround:**

Oxitropium bromide (OB) and formoterol fumarate dihydrate (FFD) are inhaler molecules that are widely used in the treatment of chronic lung diseases.

**Objective:**

The goal of this work was to create a reversed phase–ultra performance liquid chromatography (RP-UPLC) technique for assay and identification of OB and FFD, as well as identification and estimate of its associated compounds in pressurized metered dose inhaler product (pMDI).

**Method:**

Separation of oxitropium and formoterol peaks were enhanced on a C18 (50 × 2.1 mm × 1.7 μm) UPLC column with ethylene-bridged-hybrid technology, The mobile phase consists of buffer (0.07 M KH_2_PO_4_) and acetonitrile (80:20, *v/v*). The detector wavelength of 210 nm, flow rate of pump 0.6 mL/min, and oven temperature for column were set at 25°C. The injection volume was 10 μL. The method run time was 2 min. The mobile phase was used as the solvent.

**Results:**

Retention times (RTs) were 0.5 min for OB and 1.0 min for FFD. The assay analysis was linear range for all analytes within the range for concentrations 0.03—14.8 µg/mL of OB, 0.01–0.88 µg/mL of FFD. LOD values and LOQ values 0.009 and 0.026 µg/mL for OB and 0.003 and 0.009 µg/mL for FFD, respectively. Recoveries were obtained at 96.3% for OB and 97.2% for FFD. Precisions values were (as RSD, %) ≤1.5%.

**Conclusions:**

With the UPLC method developed and validated according to the current ICH guidelines, it is possible to simultaneously detect OB and FFD of assay analysis in pMDI products accurately, precisely and selectively, independent of the matrix effect.

**Highlights:**

The present method is the first method in the literature based on the UPLC method for this purpose. The UPLC method is a time-saving method, it provides a faster and cheaper technique than the high performance liquid chromatography (HPLC) method.

Chronic obstructive pulmonary disease (COPD) is one of the world’s top three killers, with low- and middle-income countries (LMICs) accounting for 90% of all deaths (1). In 2012, COPD killed around 3 million people, accounting for 6% of all deaths globally. COPD is a significant public health issue that is both preventable and curable (2). COPD is currently the fourth greatest cause of death worldwide, with the World Health Organization (WHO) projecting that it will become the third major cause of death by 2030 (3).

Bronchodilator medications are frequently used in the treatment of asthma and COPD. Both sympathetic system activation and parasympathetic system inhibitory processes are used to sustain bronchodilation. Bronchodilator therapy in COPD did not change significantly. After the emergence of new ultra-long-lasting bronchodilator medicines in recent years, a single-dose inhaler treatment per day in the management of stable COPD has become a topical concern (4). COPD treatment now includes combinations of two long-acting adrenergic agonists (LABAs) and long-acting muscarinic antagonists (LAMAs). Because activating two adrenergic receptors has a signaling route distinct from antagonizing the M3 receptor, and it generates inhibitory effects on inflammatory cells, these dual LABAs may have synergistic anti-inflammatory effects, although these anti-inflammatory effects in COPD patients have not been clearly demonstrated (5).

The inhaled anticholinergics approved for clinical use are ipratropium bromide, oxitropium bromide (OB), and tiotropium bromide. The first GOLD Report identified two short-acting anticholinergics in common usage at the time: ipratropium bromide and OB (Table 2). These two drugs remain the sole SAMAs included in the 2020 report. Tiotropium was added in 2003, aclidinium bromide and glycopyrronium bromide in 2013, umeclidinium in 2016, and glycopyrrolate and revefenacin in 2020 (6). Formoterol fumartate (FF) is a long-acting B2 agonist (LABA) used as a bronchodilator in the treatment of asthma and COPD. In the treatment of COPD, drug combinations showing LABA and COPD effects have been used together. OB and FF (Figure 1) ingredients are in the European Pharmacopoeia 10.0.

**Figure 1.**
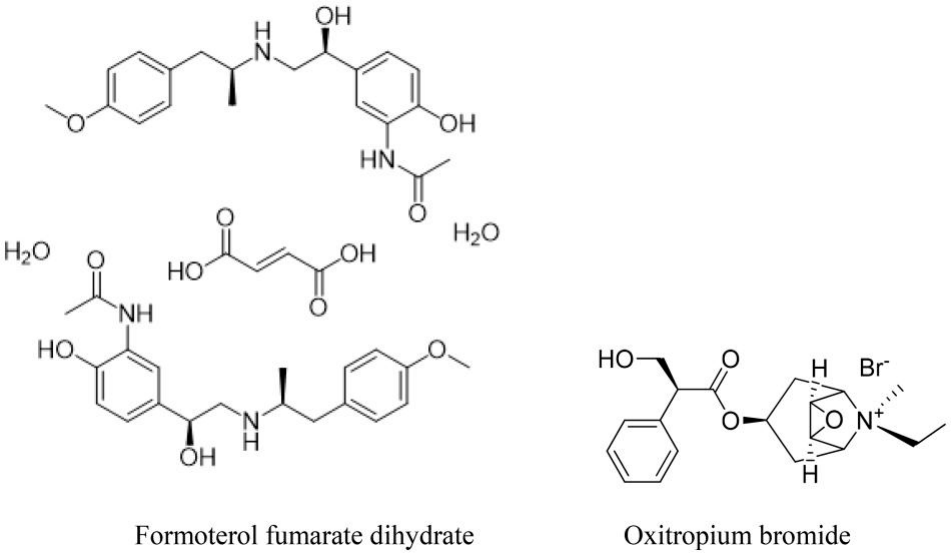
Chemical structures of FFD and OB.

**Table 2.** Purity results of the Formoterol and Oxitropium

Solution	Compound	Purity angle	Purity threshold	Result
Standard	Oxitropium	0.372	0.601	Pass
	Formoterol	2.118	3.086	Pass
Sample	Oxitropium	0.373	0.596	Pass
	Formoterol	2.039	3.192	Pass

Inhaled bronchodilator medications are classified as either short-acting (SABA) or long-acting (LABA) based on their length of action. SABA drugs are used “when necessary” during periods of increased symptoms due to the rapid onset of their effects, whereas LABA drugs are used regularly and for symptom control (7). Bronchodilator drugs are divided according to their mechanism of action: beta-2 agonists and anticholinergics. Formoterol fumarate dihydrate (FFD) is a LABA drug active ingredient. OB is a SABA drug active ingredient. SABAs are widely regarded as the first-line treatment for asthma. SABA is also recommended as the first line of treatment for COPD patients by the National Institute for Health and Clinical Excellence (NICE).

A review of the literature indicated that numerous methods for determining LABAs and SAMAs were described. Ipratropium and albuterol have been studied as combinations of beta-2 agonists and anticholinergic drug active ingredients (8). A breath-triggered device was loaded with fluticasone propionate and formoterol fumarate (FF). Image analyses were performed, and a gamma camera was employed to obtain anterior and posterior two-dimensional images of drug buildup, in contrast to usual procedures (9). Tiotropium bromide and ciclesonide were analyzed in another FF combination (10). Pharmacokinetics of three doses of budesonide, glycopyrronium, and FFD compared with active controls in pressurized metered dose inhaler product (pMDI) validated high performance liquid chromatography (HPLC)-MS/MS determined using the methodology (11). The pharmacokinetics of two (budesonide and formoterol) inhalations conducted a study on combinations in asthma patients. For each medication, two independent LC-MS/MS techniques were designed and used (12). Another study was conducted on the combination of salmaterol/formoterol and fixed-dose formoterol/budesonide used in combination therapy with inhaled corticosteroid and LABA in a single inhaler (13). The reported analytical method for OB assay is a capillary electrophoretic method with a run time of 150 min, in which OB is used as an internal standard (14). The reported analytical methods for assay of FF is ultra performance liquid chromatography (UPLC) method with a run time of between 5 and 25 min. (15). There are also studies conducted with the UHPLC-MS/MS system only for formoterol (16). In the literature, no study was found in which both actives were found together. There are publications containing separate analyzes of both actives substances. However, there is no combined analysis method for both active substances. The proposed method is the first method for this purpose. The developed method will be useful to analyze both actives together and quickly in routine quality control analysis and product development studies a well separation in the drug products.

## Experimental

### Apparatus

- The UPLC studies were performed on a Waters^®^ ACQUITY system (Milford, MA, USA) that has a PDA detector, a cooling autosampler, and an oven temperature control. For data collection, chromatographic equipment coupled to Waters Empower 2 software was employed.
- Weighing analytical balance (Mettler Toledo XP3U Ultra Micro Balance, Switzerland).
- Sonicator (Bandelin sonorex ultrasonic cleaner, Germany).
- BFC filtration unit with a vacuum pump (BF-S2500, Abel Industries, Ltd, Canada) Reagents.
- OB working standards were arranged by DEVA Holding AŞ, Turkey, and FFD working standards were arranged by Melody Healthcare Pvt. Ltd, India.
- Potassium dihydrogen phosphate dihydrate from Merck, GMBH, Germany.
- HPLC grade acetonitrile from Merck, GMBH, Germany.
- Elga Purelab Flex 2 (France) was used to gather purified water. As membrane syringe filters, PVDF filters with 0.2 µm properties were obtained from Merck.

### Preparations of Standard Solution

Preparation of OB stock standard solution: 10.0 mg of OB standard was weighed into a 100.0 mL measuring flask. A total of 70 mL of diluent was added. It took 5 min of sonication to dissolve. Dilute to volume with diluent (C_OB_ ≅ 100 µg/mL). Preparation of FFD stock standard solution: 12.0 mg of FFD standard was weighed into a 100.0 mL measuring flask. A total of 70 mL of diluent was added. It took 5 min of sonication to dissolve. Dilute to volume with diluent; 2.5 mL of this solution was transferred to a 50.0 mL measuring flask and diluted with diluent to volume (C_FFD_≅ 6 µg/mL).

Transfer to 5.0 mL of OB stock standard solution and 5.0 mL of FFD stock standard solution into a 50.0 mL measuring flask. Dilute to volume with diluent (C_OB_ ≅ 10 µg/mL and C_FFD_ ≅ 0.6 µg/mL).

### Preparations of Test Solution

**Figure 2.**
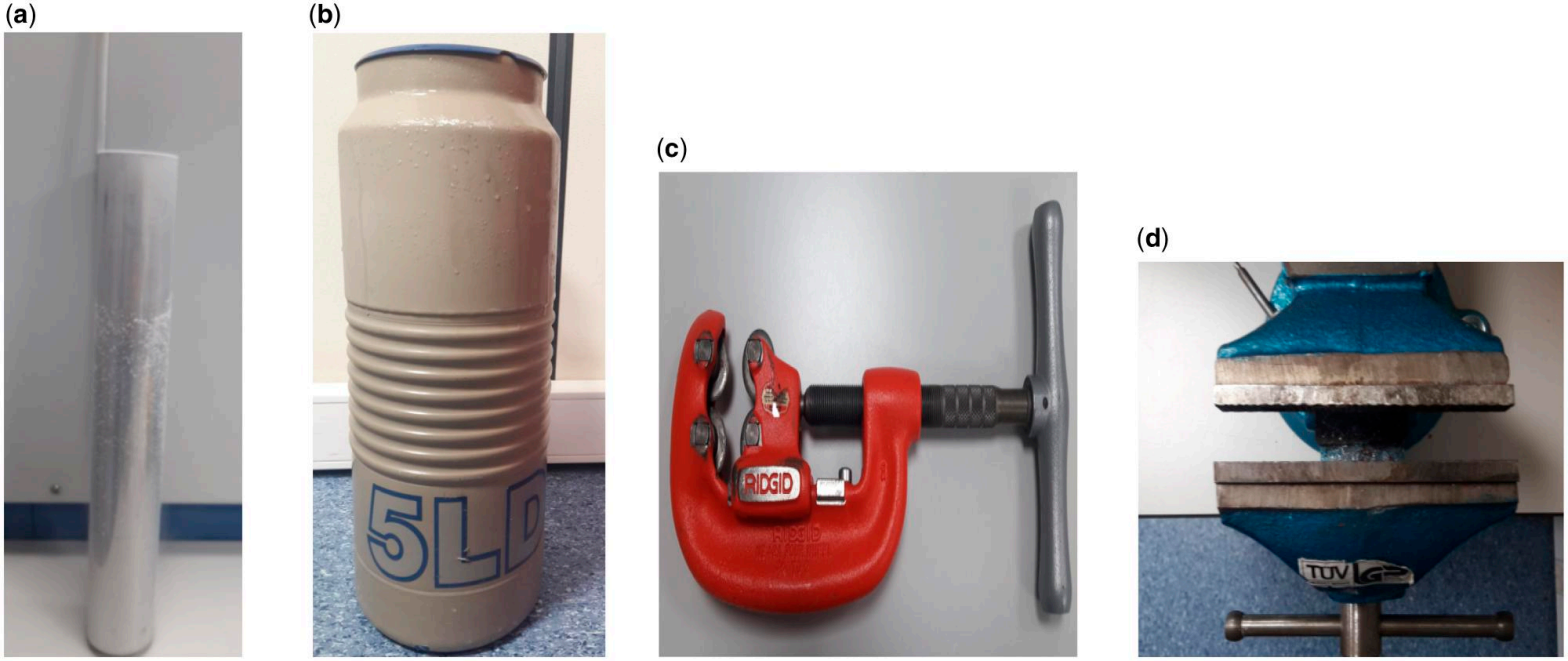
Photographs of apparatus for sampling. (A) Sample dropping apparatus into the liquid nitrogen tank. (B) Tank with liquid nitrogen. (C) Cannister cutting apparatus. (D) Compression apparatus.

All canisters were weighed in whole. The canister was placed as a valve up on the tube shown in the sample dropping apparatus (A) in Figure 2, and this tube was placed in the tank (B) filled with liquid nitrogen in Figure 2. The tube was kept in this nitrogen tank for 3–4 min. The tube was removed, and the drums were removed from this tube. Quickly cut the can from the valve neck using the apparatus shown in C and D in Figure 2. The solution was transferred to a measuring flask with a capacity of 100.0 mL. Wait until the HFA 227EA gas has completely evaporated. The box was washed with 10.0 mL of diluent, and this solution was transferred to the uniform measuring flask; 70.0 mL of diluent was added, and then sonicated in an ultrasonic bath for 5 min. Make up to volume with diluent; 7.0 mL of this solution was transferred to a volumetric flask of 100.0 mL. Dilute to get the desired volume. Finally, the empty canister was cleaned and dried. The dried canister was weighed (C_OB:_ 10.2 µg/mL, C_FFD_: 0.613 µg/mL).

#### Linearity

Each analyte was evaluated by displaying calibration curves based on the peak areas obtained from the standards with both analytes. The linearity study were prepared for assay analysis. The concentration was between 0.026 and 15 µg/mL for OB and between 0.009 and 0.9 µg/mL for FFD.

#### Specificity

No interference from the ambient matrix (impurity, placebo, and solvent) was observed in the retention times (RTs) of both active ingredients, proving the specificity of the method. Noninterference of placebo, impurity, and blank peaks at the chromatographic RT of oxitropium peak and formoterol peak were the evidence for method specificity.

#### LOD and LOQ

LOD and LOQ were calculated using the following equations;

(σ): The standard deviation in the lowest concentration

(S): The slope of the calibration curve

$$\mathrm{LOD}=\frac{3.3\sigma }{S}$$

$$\mathrm{LOQ}=\frac{10\sigma }{S}$$

The standard deviation of technique repeatability was used to measure precision. This is calculated from the n number of determination results. Six test solutions were prepared. The concentrations were 10 and 0.6 µg/mL of OB and FFD, respectively. The tests have been completed by the same analysts, *n* times, and under similar conditions as possible. Loss on drying must be determined only once, and reference and reagent solutions must be prepared once. The absolute standard deviations of results obtained from *n* number of test solutions and relative standard deviations are calculated as given below.

The calculation of absolute standard deviation:

$$\mathrm{Sabs}=\sqrt{\frac{\sum {\left(xi-\mathrm{xavg}\right)}^{2}\;}{n-1}}$$

where, *xi* = Individual results; *xavg* = The average of all individual results; *n* = Number of measurements; *Sabs* = Absorbance of treated solution.

The calculation of relative standard deviation:

$$\mathrm{RSD}\%=\mathrm{Sabs}\;*\;\frac{100}{\mathrm{xavg}}$$

#### Accuracy and recovery

For testing the accuracy of the method the practice will be conducted by different test solutions on three concentrations which are LOQ, 100% and 150% by taking the specification level as 100%. For all levels, 3 test solutions were prepared. Solutions were prepared to add OB and FFD standards; LOQ level, 100% and 150% concentrations, to the placebo mixture. Preparation of accuracy solution at LOQ, 100% and 150% concentration.

#### Robustness

Robustness studies were completed at three different flow rates as (0.5, 0.6, and 0.7) mL/min, 20, 25, and 30°C for column furnace temperature change, and 80:20, 75:25, and 85:15 (*v/v*) for mobile phase buffer ratio change.

## Result and Discussion

### Method Development

This study was started with a UPLC BEH C_18_ column for the mobile phase (phosphate buffer: acetonitrile) at a ratio of 50:50 (*v/v*), a flow rate of 0.5 mL/min, and a column temperature of 25 ± 5°C. The pH of the phosphate buffer used was measured as 4.6 ± 0.05 without the addition of any acid or base. The resolution and tailing factor were evaluated in the mobile phases obtained by mixing with acetonitrile or methanol at different ratios. When methanol was used instead of acetonitrile for the organic phase, more methanol was needed to shorten the RTs. Selecting acetonitrile as an organic solvent shortened the run time and minimized organic solvent consumption. To optimize the RTs and peak shapes, the organic solvent ratio was increased from 50 to 80%. Both the RTs and the tailing factor of the formoterol peak decreased as the concentration increased. Similarly, the RT decreased for the oxitropium peak. The optimum solvent ratio was determined considering the RTs of the peaks and tailing factors before validation. C_8_ and C_18_ columns of the same length and diameter were used. It was observed that the two interested peaks were not separated from each other with C_8_ columns. After a very long analysis time (25 min) for UPLC, a separation of approximately 22 min for formoterol peak and approximately 4 min for oxitropium was obtained. To shorten the analysis time, the flow rate was raised from 0.5 to 0.6 mL/min. After trials on the chromatographic conditions, optimum conditions have been determined.

### Method Validation

The technical requirements of International Conference on Harmonization (ICH) guideline (17) verified method validation of UPLC method for OB and FDD in pMDI. Method validation parameters were linearity, precision, accuracy, recovery, robustness, detection, and quantitation limits. LOD and LOQ levels were also determined so that they can be used in other quantification studies in pMDI products (NGI and DUSA analyses).

### Linearity

Linearity study were prepared at LOQ, 10, 20, 50, 80, 90, 100, 110, 120, and 150% concentration levels by the serial dilution by standard solutions. Table 1 shows the R and R^2^ values obtained from the solutions prepared for the linearity study.

**Table 1.** Linearity and sensitivity of FFD and OB

Compound	Linear regression equation	R^2^	LOD, µg/mL	LOQ, µg/mL
Formoterol fumarate dihydrate	*y* = 63175505,3*x* - 25,7	0.9999	0.003	0.009
Oxitropium bromide	*y* = 14413215,4*x* - 56,1	1.0000	0.008	0.026

### Specificity

**Figure 3.**
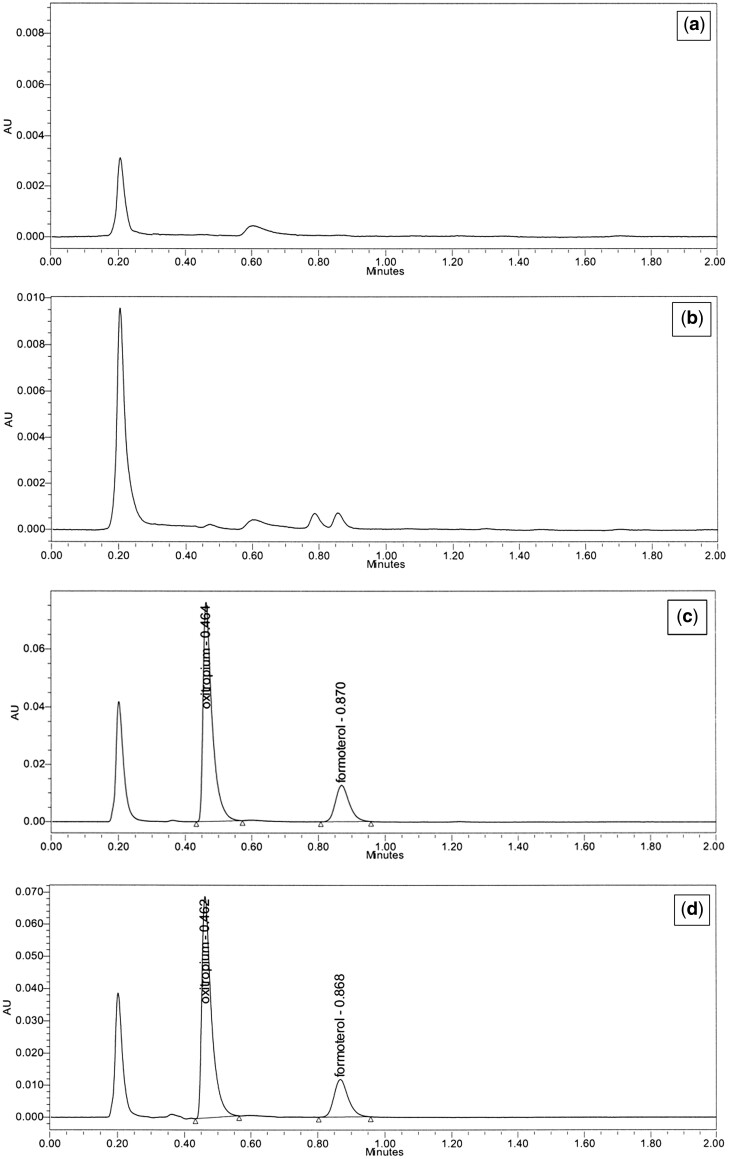
(a) Chromatogram of blank. (b) Chromatogram of plasebo. (c) Chromatogram of standard. (d) Chromatogram of test sample.

No peak interference was observed at the output times of both active substance peaks. Moreover, it was determined that there was no spectral interference from the matrix for both the oxitropium and formoterol peaks. The same concentrations were prepared for both standard and test solutions, blank solution, and treated solution without both active substances (placebo solution). These concentrations are 10 µg/mL for OB and 0.6 µg/mL for FFD. Specificity test solutions (Figure 3a–d) were run between 200 and 400 nm. Injections were obtained from the UPLC system with PDA. The peak purity angle and purity threshold data for both active substances were evaluated. Since purity angle < purity threshold, the active substance peaks are specific. The results are given in Table 2.

### Accuracy

Recovery percentages were determined in the accuracy study. Both test solutions and standard solutions were injected into a UPLC system. The results are given in Table 3. The swystem precision area was tested using the test solutions data obtained. The results are given in Table 4.

**Table 3.** The average recoveries of FFD and OB (data are means ± SD, n = 6)

Compound	Concentration, µg/mL	Recovery, %	RSD, %
Formoterol fumarate dihydrate	0.009	92.4	0.042
	0.608	99.6	0.006
	0.910	99.6	0.003
Oxitropium bromide	0.027	90.1	0.041
	10.223	99.2	0.003
	15.373	99.5	0.004

**Table 4.** Precision of the assay of FFD and OB (based on assay of six replicates of samples on different days)

Compounds	Concentration (mg/canister)	Intra-day precision RSD, %	Inter-day precision RSD, %
Formoterol fumarate dihydrate	0.790	2.1	0.9
Oxitropium bromide	13.712	1.5	0.1

### Method Robustness

The column oven temperature was set as 20, 25, and 30°C, and 100% standard solution was injected to the system. The column temperature results are shown in Table 5. Different flow rates of the mobile phase were set as (0.5–0.6–0.7 mL/min); 100% standard solution was injected into the UPLC system. All results are shown in Table 6. The buffer/acetonitrile ratio in the mobile phase was set from 80:20 to 75:25 and 85:15. All prepared solutions were injected. The organic ratio change of the mobile phase results are shown in Table 7.

**Table 5.** Robustness of the assay of FFD and OB based on assay of six replicates of samples on different temperatures (°C)

	RSD, %			Tailing factor		
Temperature Compounds	20°C	25°C	30°C	20°C	25°C	30°C
Oxitropium Bromide	0.1	0.0	0.2	2.0	2.0	2.0
Formoterol Fumarate Dihydrate	0.3	0.1	0.2	1.3	1.3	1.3

**Table 6.** Robustness of the assay of FFD and OB based on assay of six replicates of samples on different flow rates (mL/min)

	RSD, %			Tailing factor		
Flow rate Compounds	0.5 mL/min	0.6 mL/min	0.7 mL/min	0.5 mL/min	0.6 mL/min	0.7 mL/min
Oxitropium Bromide	0.1	0.0	0.1	2.0	2.0	1.9
Formoterol Fumarate Dihydrate	0.3	0.1	0.2	1.3	1.3	1.3

**Table 7.** Robustness of the assay of FFD and OB based on assay of six replicates of samples on organic ratio change in mobile phase (*v/v*)

	RSD, %			Tailing factor		
Mobile phase ratio Compounds	75:25	80:20	85:15	75:25	80:20	85:15
Oxitropium Bromide	0.9	0.0	0.1	2.0	2.0	1.6
Formoterol Fumarate Dihydrate	0.3	0.1	0.2	1.9	1.3	2.2

## Conclusions

With the UPLC method developed and validated according to the current ICH guidelines, it is possible to simultaneously detect OB and FFD in pMDI products accurately, precisely, and selectively, independent of the matrix effect. The present method is the first method in the literature based on the UPLC method for this purpose. The UPLC method is a time-saving method, and it provides a faster and cheaper technique than the HPLC method. This method has been found suitable in the development of pharmaceutical products containing OB and FFD in both QC and R&D laboratories.
